# Foam Glass Lightened Sorel’s Cement Composites Doped with Coal Fly Ash

**DOI:** 10.3390/ma14051103

**Published:** 2021-02-26

**Authors:** Adam Pivák, Milena Pavlíková, Martina Záleská, Michal Lojka, Anna-Marie Lauermannová, Ivana Faltysová, Ondřej Jankovský, Zbyšek Pavlík

**Affiliations:** 1 Department of Materials Engineering and Chemistry, Faculty of Civil Engineering, Czech Technical University in Prague, Thákurova 7, 166 29 Prague 6, Czech Republic; adam.pivak@fsv.cvut.cz (A.P.); milena.pavlikova@fsv.cvut.cz (M.P.); martina.zaleska@fsv.cvut.cz (M.Z.); 2 Department of Inorganic Chemistry, Faculty of Chemical Technology, University of Chemistry and Technology, Technická 5, 166 28 Prague 6, Czech Republic; Michal.Lojka@vscht.cz (M.L.); Anna-marie.Lauermannova@vscht.cz (A.-M.L.); Ivana.Faltysova@vscht.cz (I.F.); Ondrej.Jankovsky@vscht.cz (O.J.)

**Keywords:** construction composites, Sorel’s cement, foamed glass, coal fly ash, hygrothermal performance, structural parameters

## Abstract

Lightweight Sorel’s cement composites doped with coal fly ash were produced and tested. Commercially available foam granulate was used as lightening aggregate. For comparison, reference composites made of magnesium oxychloride cement (MOC) and quartz sand were tested as well. The performed experiments included X-ray diffraction, X-ray fluorescence, scanning electron microscopy, light microscopy, and energy dispersive spectroscopy analyses. The macro- and microstructural parameters, mechanical resistance, stiffness, hygric, and thermal parameters of the 28-days matured composites were also researched. The combined use of foam glass and fly ash enabled to get a material of low weight, high porosity, sufficient strength and stiffness, low water imbibition, and greatly improved thermal insulation performance. The developed lightweight composites can be considered as further step in the design and production of alternative and sustainable materials for construction industry.

## 1. Introduction

The rapid increase in the world’s population, together with higher demands on living standards, results in the growth of global urbanization. While in 1950 only 30% of the world’s population resided in urban areas, by 2018 around 55% of the global population was urban. The United Nations [1] further expects a further increase by 2050 due to the continuing urban growth, especially in developing countries. Herewith, the total global production of construction materials in 2017 was 17.5 billion tons which was more than twice that reported in 2000 [2]. This growth goes hand in hand with higher demand for building materials and the burden caused by the consumption of non-renewable materials, use of energy during manufacture and greenhouse gas emissions which need to be mitigated [3,4,5]. According to the reports of IEA and UN [6], the building and construction industry used 125 EJ of energy and produced 9.7 Gt of CO_2_ emissions in 2018. Nearly a third of the total CO_2_ emissions produced by the construction industry comes from the manufacture phase of building materials [6], where silicate-based materials are the most used today [2]. Portland cement (PC) concrete with its applicability, formability, advanced properties, and low manufacture cost represents the typical material of this group [7,8]. The main disadvantage of concrete is the huge CO_2_ emissions from fuel combustion to ~1450 °C and calcination reactions during PC production [9,10]. Thus, the environmental burden related to the production of building materials cannot be ignored and the development of new or improvement of existing materials which will serve as environmentally attractive alternatives to commonly used materials is needed.

Recently, there is a new increasing trend towards the use of Sorel’s cement composites in the building industry [11,12,13,14,15,16,17] and stabilization of fly ash from municipal solid waste and waste water sludge incinerators [18,19]. Sorel’s cement, also known as magnesium oxychloride cement (MOC), belongs to a wide group of magnesia-based binders [20]. These binders are composed of a concentrated aqueous solution of MgCl_2_ and light-burned MgO, and may form four crystalline phases depending on temperature during solution reaction. Phases 2 (2Mg(OH)_2_·MgCl_2_·4H_2_O) and 9 (9Mg(OH)_2_·MgCl_2_·5H_2_O) are stable at temperatures above ~100 °C while phases 3 (3Mg(OH)_2_·MgCl_2_·8H_2_O) and 5 (5Mg(OH)_2_·MgCl_2_·8H_2_O) are stable at temperatures below ~100 °C [21,22,23].

The reason for the increased interest in MOC are its unique properties, such as high mechanical strength and stiffness, resistance to fire and abrasion, or filler bonding ability [24,25,26,27], and advantages which can be in some cases preferable or superior to properties of commonly used PC. One of the advantages is the rapid precipitation reaction of MOC, the fast formation of phase 3 and phase 5 crystals, and the associated rapid increase of material strength. Previous studies describing the development of phase 3 and phase 5 in the reaction process show the progress of their formation. The stoichiometric phase 3 is fully formed (>99 wt.% of the sample) after only 36 h. The study of formation of the phase 5 described the formation process similarly, determining the formation time of the pure phase 5 (>99 wt.% of the sample) to 96 h [28,29]. Concerning the strength development, preceding research has shown the significant increase in the compressive strength in the first seven days of curing. The value of compressive strength after this period did not increase much more, showing the material has fully developed [30,31,32]. Another advantage of MOC is its lower environmental impact during the life cycle compared to PC. Production of light-burned MgO can occur through calcination of magnesite (MgCO_3_) or dolomite (CaMg(CO_3_)_2_) or synthetically from magnesium brines or seawater. Although the calcination temperature is lower than calcination temperature of PC (~750 °C vs. ~1450 °C), some studies show that the total CO_2_ emissions only from calcination is higher due to higher volume of raw materials, higher quantity of combusted fuel and released CO_2_ during material decomposition [33,34]. On the other hand, MOC-mortar has the ability to carbonate during long-time curing period and absorbs CO_2_ from the atmosphere. Although some studies highlight the potential of this ability to classify MOC as low-carbon or carbon-neutral alternative material, only a few studies examined this aspect further, because of a very slow carbonation process in the natural environment [34,35]. Recent research evaluated that the amount of CO_2_ absorbed by MOC-mortar is 20–40% of the initial CO_2_ emissions in 15 years [36]. Nevertheless, the total environmental impact of MOC can be considered lower than the impact of PC [33].

Unfortunately, a big disadvantage of MOC-based materials and limitation for various uses is their poor water resistance [20]. When immersed in water, the crystalline phases dissolve, which results in increasing porosity and loss of strength [37,38]. It is known that water resistance can be increased using additives such as organic and inorganic agents and acids [39,40,41], microfillers and nano-additives [42,43,44]. Also, some of these microfiller additives are produced as waste materials and their incorporation into MOC-composite increase its ecological value.

Consumption of raw materials is another environmental impact of building material production which cannot be overlooked. A standard concrete mix is composed of approx. 80–85% of natural raw aggregate depending on the targeted material characteristics [45]. With the global use of concrete amounting up to 20 billion tons annually [46], consumption of aggregate could be around 16 to 17 billion tons. Although natural raw aggregates are part of non-critical raw materials [47], extraction of these materials has an impact on the environment and landscape [48]. To reduce the consumption of raw aggregates, alternative aggregate must be used in building materials. As mentioned earlier, MOC-based composites possess good bonding ability which is applicable for use of a wide range of raw or waste materials as aggregate or fillers. Many recent studies were focused on an improvement of thermal properties of MOC-composite with lightweight materials. For instance, Zhou et al. [21] and He et al. [49] incorporated waste wood into MOC which reduced thermal conductivity and increased flexural strength, but also increased water absorption. Záleská et al. [39] investigated MOC with waste expanded polypropylene aggregate which reduced heat transfer as well as mechanical strength of final material. Waste tire rubber used by Biel and Lee [50] or Pavlíková et al. [51] resulted in a huge decrease of mechanical properties.

As we can see from the studies conducted, depending on alternative materials and their dosage, some properties will increase while others can be dramatically reduced. The same effect is observable with additives mentioned in the earlier paragraph, a dosage of fly ash in MOC-mortar resulted in improved workability, reduced porosity and water resistance due to the formation of M-S-H gel, but the higher content of fly ash in composite mixture reduced mechanical strength a stiffness [42,52]. Therefore, it is crucial to find a balance between the type of alternative aggregate, its dosage and required functional and technical properties of the manufactured materials.

The goal of this study was to develop new MOC-based lightweight composite material with alternative aggregate to standardly used natural sand. Considering previously reported studies, foam glass gravel was applied as aggregate. It is a material with high strength, limited heat transport parameters, and low water permeability. To the best authors’ knowledge, there are no published studies on the production of lightweight MOC composites with foam glass. Moreover, to improve the functional and technical parameters of produced composites, fly ash was batched as partial substitute of glass granulate. Although the usage of fly ash was due to the promising results on structural and mechanical parameters of fly ash-doped MOC composites published recently [42,52,53,54], the MOC-fly ash composites were only scantly studied to date. In this way, ternary MOC composites were designed and tested which represents novel and alternative solution for the development of advanced construction materials for construction practice. The eco-efficiency of the researched composites made of secondary raw material (foam glass) and industrial by product (fly ash) was also motivation of the presented study. The prepared materials were characterized using a variety of analytic methods aimed at the assessment of structural parameters, mechanical properties, heat transport and storage ability, and water absorption. Such broad experimental analysis of MOC lightweight composites was not presented yet and can be considered as further step towards application of these alternatives to Portland cement-based composites in building industry.

## 2. Materials and Methods

### 2.1. Materials and Preparation of Samples

For the preparation of composites, magnesium oxide (MgO) produced by the company Styromagnesit Steirische Magnesitindustrie Ltd. (Oberdorf, Austria) was used. The MgO powder was composed of MgO (81.0 wt.%), Al_2_O_3_ (6.1 wt.%), Fe_2_O_3_ (3.9 wt.%), CaO (4.8 wt.%), SiO_2_ (3.5 wt.%), and SO_3_ (0.5 wt.%). Magnesium chloride hexahydrate (MgCl_2_∙6H_2_O) of p.a. purity delivered by Lach-Ner, Ltd. (Neratovice, Czech Republic) was used in the form of aqueous solution. In the control (reference) composite signed as MOC-Sref, quartz sand coming from Filtrační písky, Ltd. (Chlum u Doks, Czech Republic) was used as the only aggregate. Quartz sand filler was prepared as a mixture of three fractions, 0.063–0.5 mm, 0.5–1.0 mm, and 1.0–2.0 mm respectively, in a weight ratio of 1:1:1. In the lightened composites signed as MOC-G, sand was fully replaced by foam glass (glass granulate) that was produced by REFAGLASS, Ltd. (Vintířov, Czech Republic). It is made of glass powder ground from the recycled glass and chemical foaming agent. Glass granulate consisted of SiO_2_ (65.8 wt.%), CaO (12.4 wt.%), Al_2_O_3_ (4.7 wt.%), MgO (2.3 wt.%), Fe_2_O_3_ (0.5 wt.%), K_2_O (0.9 wt.%), SO_3_ (1.2 wt.%), and Na_2_O (12.0 wt.%). As the foam glass was originally delivered in 0–4 mm fraction, it was sifted through the standardized stainless steel sieve to get fraction 0–2.0 mm. To produce the lightweight composites more eco-friendly, coal fly ash (Opatovice Inc. thermal power plant, Opatovice, Czech Republic) was applied as a partial volumetric substitute of foam glass. In this way, the ternary mixture of MOC, foam glass, and fly ash was obtained. The substitution ratio of foam glass by fly ash was 5%, 10%, and 15% by volume. In fly ash, following major oxides were present: SiO_2_ (51.9 wt.%), Al_2_O_3_ (34.3 wt.%), Fe_2_O_3_ (6.8 wt.%), CaO (1.8 wt.%), K_2_O (1.7 wt.%), TiO_2_ (1.5 wt.%), MgO (1.4 wt.%), SO_3_ (0.4 wt.%). As specified in ASTM C618 [55], the used fly ash was classified as F type, possessing pozzolanic properties. The composition of the examined composites is summarized in Table 1. In case of composites with foam glass, 1/3 of batch water was used for wetting of the lightweight aggregate, which was done 24 h before samples mixing. The remaining batch water (567.25 g) was used for the preparation of MgCl_2_ solution.

Portland blended fly ash cement and natural pozzolan Portland cement used in the construction industry contain natural radioactive isotopes, such as radium (^226^Ra), thorium (^232^Th) and potassium (^40^K), and their radioactivity may exceed the maximum safe value [56]. Typically, activity concentration of radionuclides in coal fly ash is higher in finer particles [57,58]. In fly ash used in this study, presence of radium and thorium was not confirmed. The only potential radioactive element present in fly ash was potassium with the mass concentration of 1.4%. Taking into account fact that the radioisotope ^40^K composes of approx. 0.012% of natural potassium, and low concentration of potassium in MOC-GF mixtures (maximum 4 g of natural potassium, which represents ~0.0005 g of radioisotope ^40^K), the use of fly ash in the composition of the researched composites does not represent any problem for their natural radioactivity. Moreover, as reported, e.g., in [34,59], the MgO radioactivity is almost negligible compared to that of Portland cement. In the caustic magnesia used in this work, no potentially radioactive elements were detected.

In the preparation of composite mixtures, the aqueous solution of MgCl_2_ was poured into a standard mixing machine and MgO was added. This mixture was mixed for 30 s at low speed (paddle 140 rpm, mixing head 62 rpm). After that, the quartz sand or foam glass was admixed under continuous stirring (30 s). Then, the composite mixture was homogenized for another 30 s at high-speed regime (paddle 285 rpm, mixing head 125 rpm). At 90 s the mixer was stopped and the material stuck to the wall of the stainless steel stirring bowl was removed by a rubber squeegee. Finally, the mixture was mixed for 60 s at high-speed setting. In case of fly ash-doped materials, fly ash was mixed with MgCl_2_ water solution first. The fresh composite mixtures were cast into prismatic steel molds having dimensions of 40 mm × 40 mm × 160 mm and cubic molds having a side of 70 mm. The molds were filled in two layers whereas each layer was compacted for 30 s using a vibration plate. After removal of the hardened composites from the molds, they were freely stored in a laboratory at *T* = (23 ± 2) °C and *RH* = (30 ± 5)% for 28 days.

### 2.2. Conducted Tests and Analyzes

Using a standard sieve analysis prescribed in the EN 933-1 [60], grain size curves of quartz sand and foam glass were assessed. The particle size distribution of MgO and fly ash was tested by an Analysette 22 Micro-Tec plus (Fritsch, Idar-Oberstein, Germany) laser diffraction apparatus.

For fresh composite mixtures, spread diameter was measured in a flow table test. The test was performed in the accordance with the EN 1015-3 [61].

X-ray Powder Diffraction (XRD) was carried out using a Bruker D2 Phaser (Bruker AXS GmbH, Karlsruhe, Germany), a powder diffractometer with Bragg–Brentano geometry, applying CuKα radiation (λ = 0.15418 nm, U = 30 kV, I = 10 mA) and rotation (five rounds per minute). The step size was set to of 0.02025° (2θ) and the overall data were acquired in the angular range of 10°–80°. X’Pert HighScore Plus software (PANalytical, Almelo, The Netherlands) was applied to evaluate the obtained data.

Scanning Electron Microscopy (SEM) was used to study the surface morphology of foam glass and FA (Tescan MAIA3, TESCAN Brno, s.r.o., Brno, Czech Republic). The elemental composition and mapping were performed using an energy-dispersive spectroscopy (EDS) analyzer (X-Max150, Oxford Instruments, High Wycombe, UK) with a 20-mm^2^ SDD (silicon drift detector) detector and AZtecEnergy software (Oxford Instruments). The sample was set on a carbon conductive tape to ensure the conductivity of the experiments. For both SEM and SEM-EDS analysis, the electron beam was set to 10 kV with 5 mm working distance.

The specific density and dry loose bulk density of MgO, quartz sand, glass granulate, and fly ash were measured using a helium pycnometer Pycnomatic ATC (Porotec, Hofheim, Germany), graduated cylinder, and precise laboratory scales. Blaine fineness was tested by the use of a Blaine apparatus. Before the tests, the samples were dried at 105 °C.

For the 28-days matured samples, structural, mechanical, hygric, and thermal properties were investigated. The macrostructure of the hardened composites was characterized by bulk density, specific density, and total open porosity tests. The measurements were conducted for five samples of each studied composite. The dry bulk density *ρ*_b_ (kg∙m^−3^) of 70 mm cubes was measured in the accordance with the EN 1015-10 [62]. The specific density *ρ*_s_ (kg∙m^−3^) was measured on a helium pycnometry principle (see above). The total open porosity *Ψ* (%) values were determined based on the bulk density and the specific density data. Details on the experimental techniques used for the macrostructural parameter’s assessment can be found in [63,64]. The microstructure of the developed composites was researched using the Mercury Intrusion Porosimetry (MIP). Set of porosimeters of Pascal series (Pascal 140 and Pascal 440, Thermo Fisher Scientific, Waltham, MA, USA) was used in the microstructure characterization. The typical sample mass was ~1.5 g. The investigated parameters were the total pore volume, the total porosity, and the average pore diameter. Among the mechanical parameters, the flexural strength *f*_f_ (MPa), the compressive strength *f*_c_ (MPa), and the dynamic modulus of elasticity *E*_d_ (GPa) were tested. These experiments were done in compliance with the EN 1015-11 [65]. The flexural strength and the dynamic modulus of elasticity were measured on standard 40 mm × 40 mm × 160 mm prisms. In the compressive strength tests, the loading area was 40 mm × 40 mm. The ultrasound velocity test was conducted using an apparatus Pundit Lab+ (Proceq, Schwerzenbach, Switzerland). Because MOC-based materials are considered to disintegrate in water, the water absorption coefficient *A*_w_ (kg∙m^−2^∙s^−1/2^) was determined in a free water intake experiment. It represents the simplest technique for the characterization of the ability of porous materials to absorb water and transport it by capillary forces. The *A*_w_ test was conducted according to the standard EN 1015-18 [66]. The experimental arrangement of the test consisted of tank filled with water, and the material sample, water and vapor-proof insulated on four lateral sides by epoxy resin, hung on an automatic balance and immersed 3–5 mm in the water. The automatic balance allowed continuous recording of increasing sample mass. Based on measured results, the water absorption coefficient was evaluated as introduced by Fang et al. [67]. The heat transport and storage properties, such as the thermal conductivity *λ* (W∙m^−1^∙K^−1^), the thermal diffusivity *a* (m^2^∙s^−1^), and the volumetric heat capacity *c*_v_ (J∙m^−3^∙K^−1^) were measured using a commercially manufactured apparatus ISOMET 2114 (Applied Precision Ltd., Bratislava, Slovakia). The device operates on a transient impulse technique principle. In the experiments, the circular surface probe was placed on dry 70 mm cubes.

The thermal behavior of the prepared hardened samples was analyzed by Simultaneous Thermal Analysis (STA). The DTA and TG curves were recorded simultaneously on a Linseis STA PT1600 (Linseis Messgeraete GmbH, Selb, Germany) apparatus at a heating rate of 10 °C· min^−1^ in a dynamic air atmosphere (50 mL· min^−1^).

The Light Microscopy (LM) was performed by Navitar (Rochester, NY, USA) macro-optics with optical zoom up to 110× and recorded with a 2/3″digital camera (Sony, Tokyo, Japan) with a resolution of 5 Mpix. The samples were illuminated by a white LED ring light source with individually addressable segments and intensity. NIS-Elements BR 5.21.02 software (Laboratory Imaging, Inc., Prague, Czech Republic) with an Extended Depth of Focus Module (EDF) was used for imagining and analysis of the samples.

## 3. Results and Discussion

In this study, the composite materials containing MOC, foam glass and fly ash (FA) in various ratios were analyzed in terms of their phase composition, morphology, microstructure, thermal behavior, and mechanical properties. These results were compared to the properties of the reference samples containing only silica sand or foam glass as a filler, which were also prepared.

**Figure 1:**
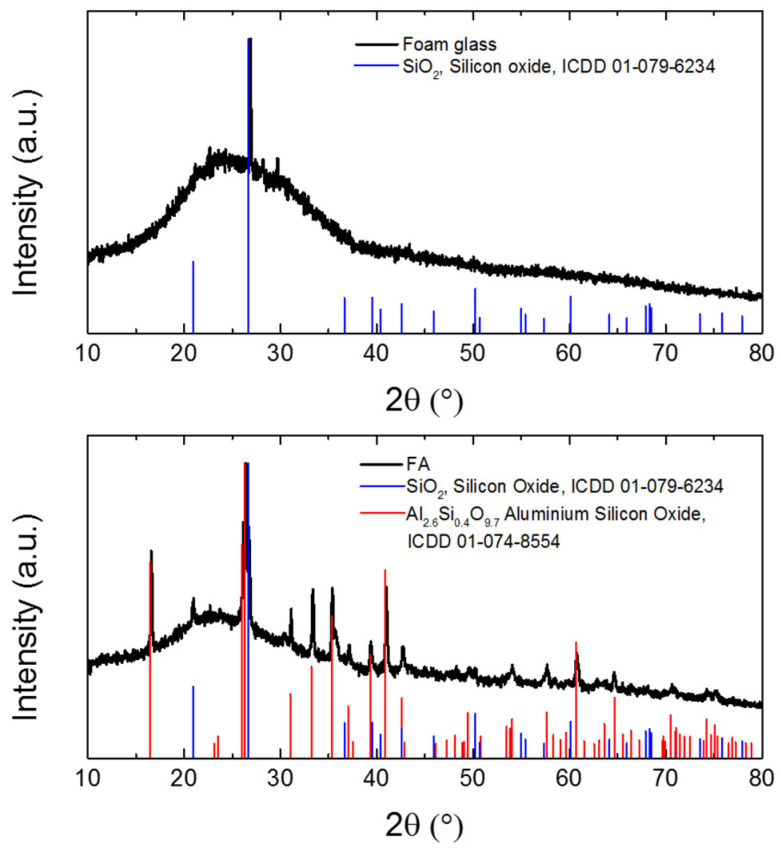
X-ray diffraction of foam glass and fly ash.

The phase analysis of the fillers was performed using XRD. The diffraction patterns of the raw materials (shown in Figure 1) provided information about the phase composition of the foam glass and fly ash. The diffraction pattern of the foam glass shows an amorphous structure with a single reflection of silicon oxide (ICDD 01-079-6234). The diffraction pattern of the fly ash presents the content of silicon oxide (ICDD 01-079-6234) and an aluminosilicate phase with the composition Al_2.6_Si_0.4_O_9.7_ (ICDD 01-074-8554). Also, this sample is highly amorphous.

**Figure 2:**
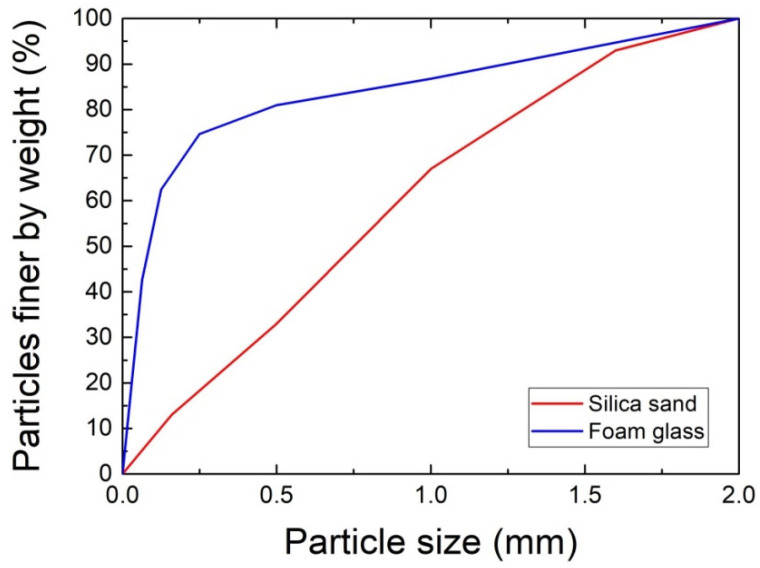
Particle size distribution curves of quartz sand and foam glass.

The grain size curves of both used aggregates, i.e., of quartz sand and foam glass, are presented in Figure 2. Both materials exhibited a similar maximum size of particles. Apparently, foam glass granules were considerably finer compared to silica sand particles.

The particle size distribution parameters of MgO and FA, which are introduced in Table 2, gave information on the high fineness of MgO powder compared to that of MgO powder. The presented particle size distribution parameters resulted in Blaine specific surface of approx. 700 m^2^∙kg^−1^, which was for FA about 40% lower. The Blaine fineness data is together with specific density and loose bulk density given in Table 3.

**Figure 3:**
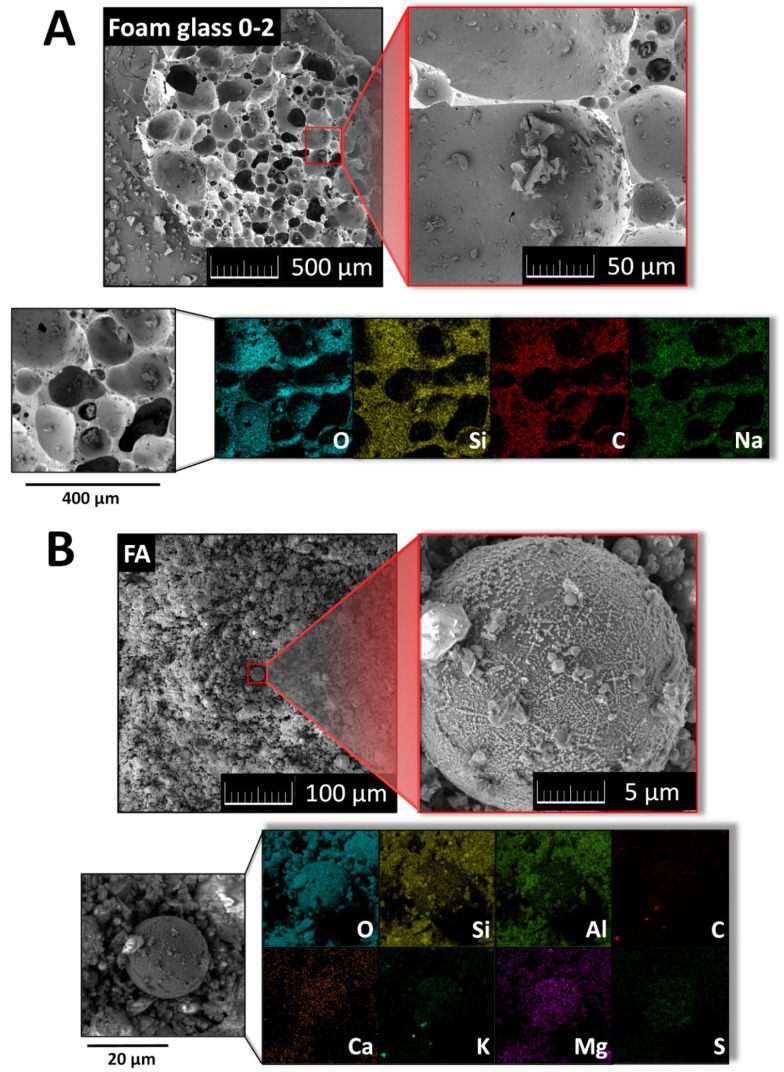
(**A**) SEM micrographs and EDS elemental maps of foam glass (fraction 0–2 mm); (**B**) SEM and EDS of FA.

The morphology of foam glass (fraction 0–2 mm) and FA was investigated using scanning electron microscopy (SEM). Elemental maps were obtained using an energy dispersive spectroscopy (EDS) analyzer (X-MaxN). Obtained micrographs of foam glass show typical structure for this type of materials, also EDS confirmed the presence of Si, O, Na and C elements (Figure 3A). Fly ash contained a high number of spherical particles with dimension between 20 to 30 μm. According to EDS, the following elements were present in the sample: O, Si, Al, C, Ca, K, Mg and S (Figure 3B). Obtained results are in good agreement with XRF data.

**Figure 4:**
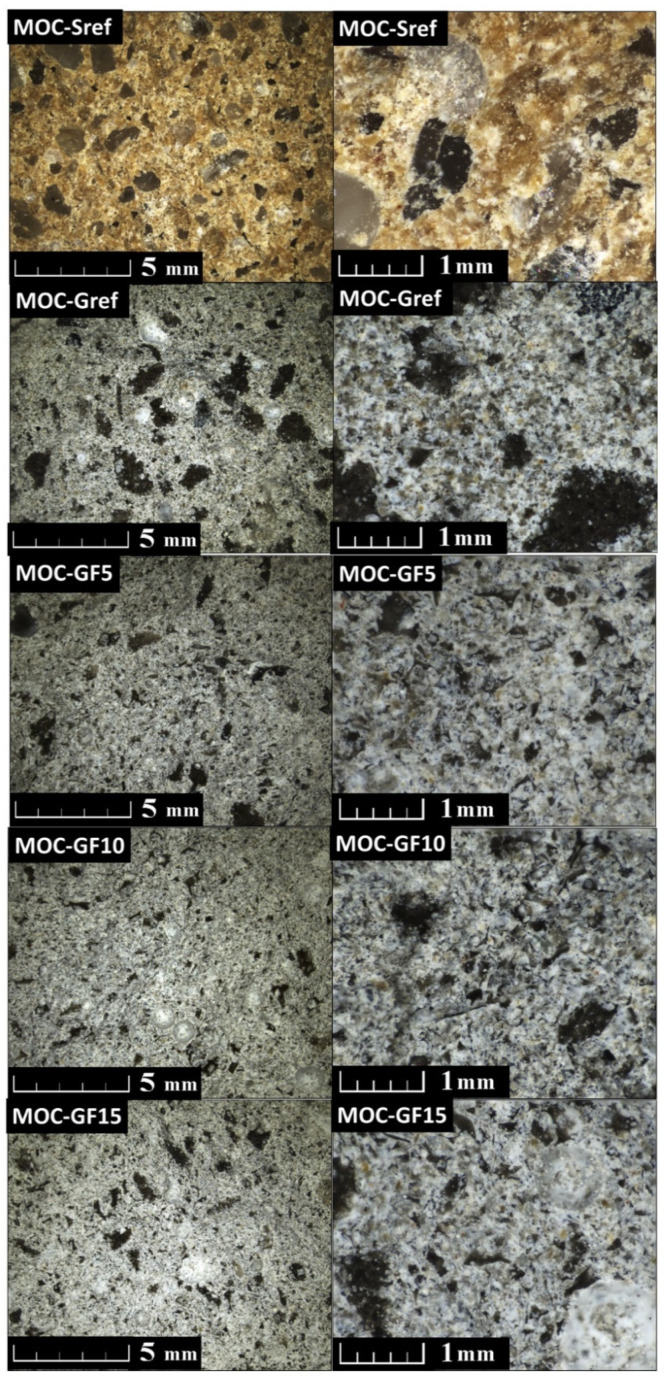
Structure of prepared composites analyzed by LM.

To study the morphology of all the prepared composites, light microscopy from fracture surface was used (see Figure 4). The samples have a compact structure with no cracks, only a few bubbles are visible. The main difference between the foam glass-fly ash composites and the reference samples is in color, which was affected by the color of the fillers. The size of the filler agglomerates differs in all samples in the range between 0.1 and 1 mm.

The macrostructural parameters determined for 28-days hardened composites are shown in Table 4. The presented data represent a mean value from the measurements of 5 samples of each composite tested. Also, spread diameter measured in a flow table test is presented. The replacement of quartz sand with glass granulate changed the consistency of the fresh composite mixtures and reduced their workability. However, it was further improved by FA admixing which was considered very beneficial for the processing of fresh mixtures, their casting into molds and compaction. The porosity of the composites with foam glass was much higher compared to the composite with quartz sand used as only aggregate. It was due to the highly porous glass granules and their low density that affected also the dry bulk density of the lightweight materials. Accordingly, the porous nature of foam glass resulted in the significant drop of specific density. In case the particle size distribution of quartz sand and FA would be similar in the whole particle size range, higher porosity and thus lower bulk density can be anticipated for the MOC/foam glass composites.

**Figure 5:**
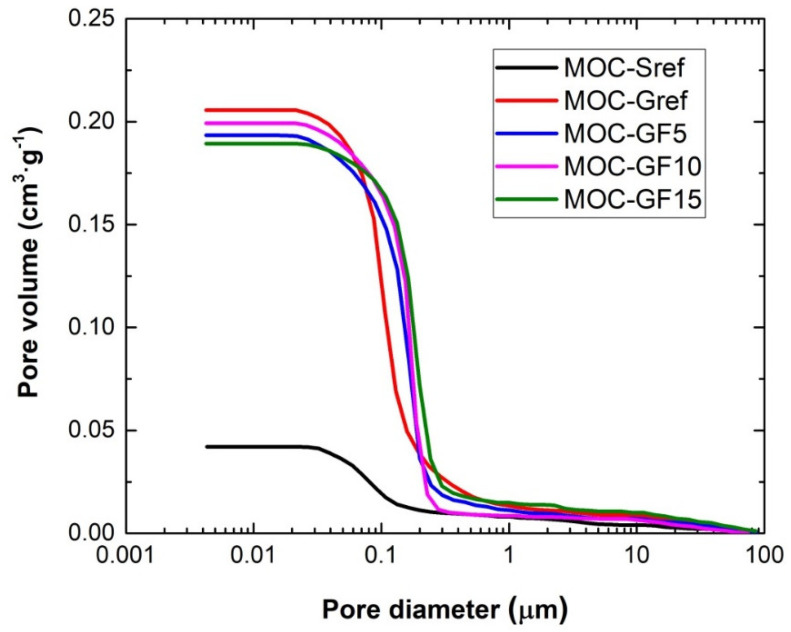
MIP cumulative pore volume curves.

**Figure 6:**
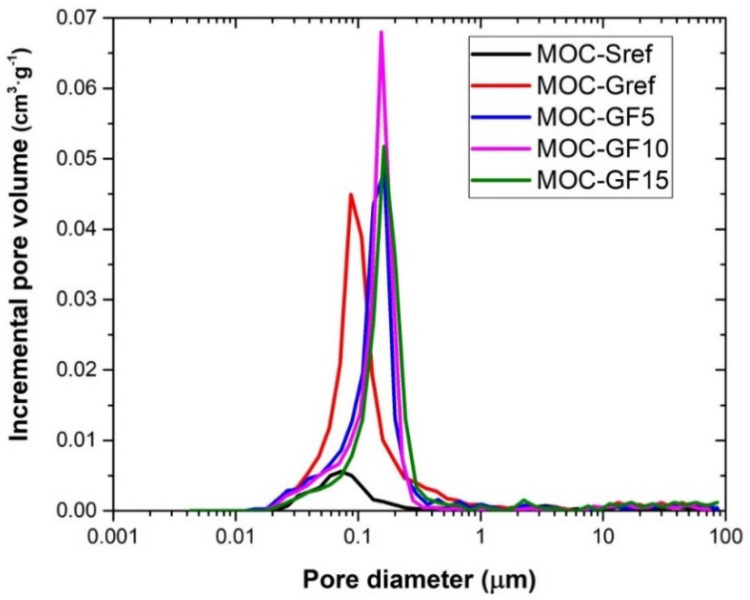
MIP incremental pore volume curves.

The cumulative pore volume curve and the incremental pore volume curve measured by MIP are plotted in Figure 5 and Figure 6. The MIP data is in good agreement with the porosity results presented in Table 4. The low volume of pores in the whole studied pore diameter range was well apparent for the reference composite MOC-Sref. The increase in pore volume using foam glass as aggregate was well documented similarly as a partial decrease in the porosity due to the filler effect of FA.

The microstructural parameters are summarized in Table 5. Both the total pore volume and Hg total porosity decreased with the dosage of FA. On the other hand, the average pore diameter was for FA doped materials higher, compared to that of MOC-Gref composite.

The flexural strength, compressive strength, dynamic modulus of elasticity and water absorption coefficient data is presented in Table 6. The reference composite with silica aggregate exhibited high mechanical resistance and stiffness such typical for MOC-based materials [68,69]. The mechanical strength and stiffness corresponded to their high porosity, i.e., these parameters were greatly reduced in comparison with the control material MOC-Sref. On the other hand, the mechanical resistance remained still high which points to the further possible lightening of these composites. Compared to ordinary cement composites, the imbibition of water into all tested materials was low. The water absorption coefficient was even 2 orders of magnitude lower than that reported, e.g., by Ma et al. who researched water absorption of cement composites with waste brick powder [70].

Parameters characterizing heat transport and storage in the developed composites are given in Table 7. These were measured for dried specimens by a transient impulse technique. The measurement accuracy of thermal conductivity was in the analyzed thermal conductivity range 10% of reading. The volume heat capacity was accessed with the accuracy 15% of reading + 1.0 × 10^3^ (J∙m^−3^∙K^−1^). The full substitution of silica sand in composites’ composition significantly improved their thermal insulation capability. It has also greatly lowered heat storage in the researched MOC-G materials. These were results of the effect of low density, thermal conductivity, thermal diffusivity, and specific heat of foam glass [71].

**Figure 7:**
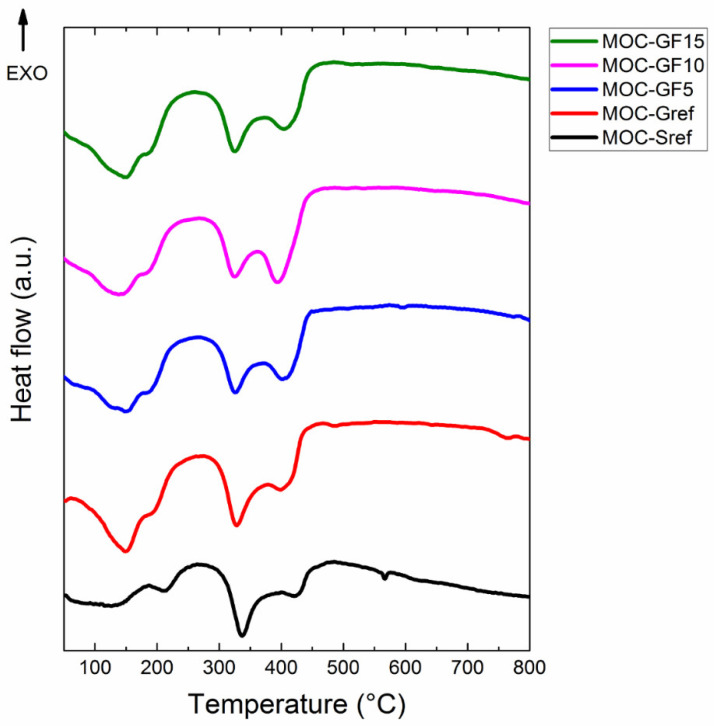
DTA curves of prepared composites measured in dynamic air atmosphere.

The simultaneous thermal analysis provided the information of the sample’s behavior while heated to 800 °C. The results can be seen in Figure 7. Four endothermic effects (with a local minimum) at 150, 200, 340 and 420 °C were detected. These endothermic effects can be assigned to the decomposition of MOC, namely the release of the crystalline water at lower temperature and hydrochloric acid at higher temperature. The decomposition of phase 5 was previously described in detail (in combination with MS) in the literature [20].

**Figure 8:**
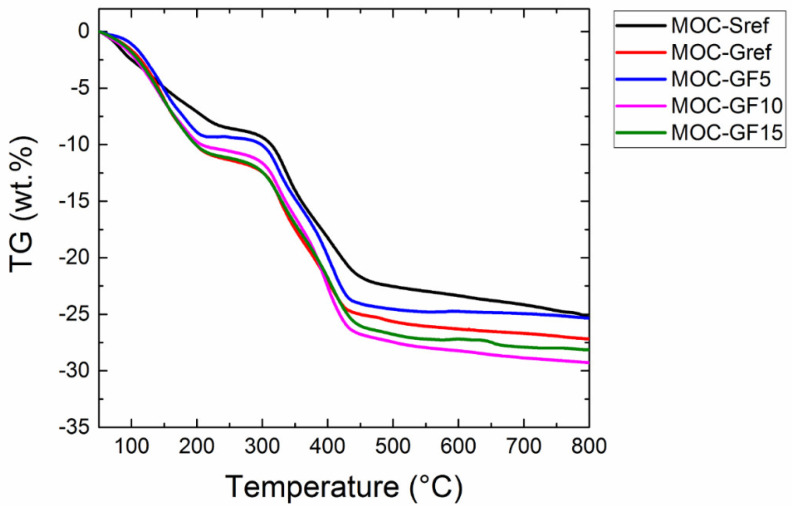
TG curves of prepared composites measured in dynamic air atmosphere.

The TG curves (see Figure 8) showed the weight decrease connected with the decomposition of MOC. The weight decrease connected to each endothermic effect agrees with the theoretical weight loss. All the samples behaved quite similarly, only the first endothermic effect of sample MOC-Sref was not as significant most probably due to lower water content. This was further approved by the TG curve, where the weight loss during this effect was smaller compared to other samples.

## 4. Conclusions

In this study, the lightweight Sorel’s cement composites were developed and tested. Following main findings were highlighted:

1) the full replacement of quartz sand by foam glass resulted in a great increase in the total porosity and pore volume, the highest porosity of 29.7% was obtained for composite made of MOC and foam glass;
2) the admixing of fly ash slightly lowered the total open porosity which decreased with the substitution ratio of glass granulate with fly ash;
3) the mechanical resistance and stiffness followed the trend of porosity, i.e., with the increase in the porosity the mechanical parameters were significantly reduced;
4) the use of fly ash in composite mix partially compensated the drop in mechanical parameters caused by the lightening of composites by foam glass;
5) the use of foam glass greatly reduced the weight of the developed composites and significantly improved their thermal insulation performance, the maximum drop in the thermal conductivity was about 75% compared to the control materials made of MOC and quartz sand;
6) as the mechanical resistance of composites with foam glass remained still high, the researched materials can be further lightened which will enable to attain materials with highly reduced heat transport ability;
7) the effect of the fly ash admixing on the hygrothermal function of the developed composites was negligible, therefore it can be simply applied as an alternative, low cost, and eco-efficient filler for MOC-based materials even in the combination with lightweight aggregate.

The broad experimental analysis of MOC lightweight composites enriched by foam glass and fly ash provided information on the functional and technical parameters of the newly produced materials and can be considered as further step towards application of these alternatives to Portland cement-based composites in building industry. Although the major motivation for the development of MOC-based materials has been driven by an environmental standpoint, the produced lightweight composites, due to their specific and unique properties, can find use in flooring systems, building envelopes, in the production of prefabricated construction systems such as facade panels, cladding insulation boards with high fire resistance, repair applications, walling blocks, etc. In future work, it will be necessary to examine durability and longtime performance of the developed composites which will enable their further modification for specific application purposes. Also, the possibility of their further lightening will be investigated in respect to the production and application technology.

## Figures and Tables

**Table 1:** The dosage of the components (g).

Composite	MgO	MgCl_2_∙6H_2_O	Water	Sand	Foam Glass	Fly Ash
MOC-Sref	1541.25	682.85	567.25	3 × 1125	-	-
MOC-Gref	1541.25	682.85	850.88	-	1564.65	-
MOC-GF5	1541.25	682.85	850.88	-	1486.42	95.54
MOC-GF10	1541.25	682.85	850.88	-	1408.18	191.07
MOC-GF15	1541.25	682.85	850.88	-	1329.95	286.61

**Table 2:** Particle size distribution parameters obtained on a laser diffraction principle.

Material	d10 (μm)	d50 (μm)	d90 (μm)
MgO	1.09	7.36	23.04
FA	23.05	42.61	67.1

**Table 3:** Basic physical properties of FA and MgO.

Material	Dry Specific Density (kg∙m^−3^)	Dry Loose Bulk Density (kg∙m^−3^)	Blaine Fineness (m^2^∙kg^−1^)
MgO	3329	839	702
FA	2057	791	403

**Table 4:** Spread diameter and basic macrostructural parameters of developed composites.

Material	Spread Diameter (mm)	*ρ_b_* (kg∙m^−3^)	*ρ_s_* (kg∙m^−3^)	*Ψ* (%)	Spread Diameter (mm)
MOC-Sref	160/165 ± 5	2127 ± 30	2430 ± 29	12.5 ± 0.3	160 × 165
MOC-Gref	150/150 ± 5	1431 ± 20	2035 ± 24	29.7 ± 0.6	150 × 150
MOC-GF5	155/150 ± 5	1453 ± 30	2041 ± 25	28.8 ± 0.6	155 × 150
MOC-GF10	155/160 ± 5	1467 ± 21	2069 ± 25	28.1 ± 0.6	155 × 160
MOC-GF15	160/165 ± 5	1495 ± 21	2056 ± 25	27.3 ± 0.5	160 × 165

**Table 5:** Microstructural parameters measured by MIP.

Material	Total Pore Volume (cm^3^∙g^−1^)	Total Hg Porosity (%)	Average Pore Diameter (μm)
MOC-Sref	0.0627	11.8	0.072
MOC-Gref	0.2057	29.6	0.087
MOC-GF5	0.1935	28.9	0.163
MOC-GF10	0.1993	28.4	0.162
MOC-GF15	0.1894	26.7	0.154

**Table 6:** Spread diameter and basic macrostructural parameters of developed composites.

Material	*f*_f_ (MPa)	*f*_c_ (MPa)	*E_d_* (GPa)	*A_w_* (kg∙m^−2^∙s^−1/2^)
MOC-Sref	23.8 ± 0.3	69.9 ± 1.0	31.2 ± 0.7	0.017 ± 4 × 10^−5^
MOC-Gref	9.3 ± 0.1	43.7 ± 0.6	10.7 ± 0.2	0.057 ± 1× 10^−4^
MOC-GF5	9.4 ± 0.1	43.9 ± 0.6	10.9 ± 0.2	0.054 ± 1× 10^−4^
MOC-GF10	9.5 ± 0.1	44.2 ± 0.6	11.2 ± 0.2	0.056 ± 1× 10^−4^
MOC-GF15	9.8 ± 0.1	45.7 ± 0.6	11.3 ± 0.2	0.059 ± 1× 10^−4^

**Table 7:** Spread diameter and basic macrostructural parameters of developed composites.

Material	*λ* (W∙m^−1^∙K^−1^)	*a* × 10^−6^ (m^2^∙s^−1^)	*c*_v_ × 10^6^ (J∙m^−3^∙K^−1^)
MOC-Sref	3.17	2.07	1.55
MOC-Gref	0.97	1.74	0.56
MOC-GF5	0.80	1.75	0.45
MOC-GF10	0.81	1.74	0.46
MOC-GF15	0.79	1.79	0.44

## Data Availability

The data presented in this study are available on request from the corresponding author. The data are not publicly available due to privacy.
